# Correlation of Panoramic Radiography, Cone-Beam Computed Tomography, and Three-Dimensional Printing in the Assessment of the Spatial Location of Impacted Mandibular Third Molars

**DOI:** 10.3390/jcm10184189

**Published:** 2021-09-16

**Authors:** Aleksandra Jaroń, Ewa Gabrysz-Trybek, Joanna Bladowska, Grzegorz Trybek

**Affiliations:** 1 Department of Oral Surgery, Pomeranian Medical University in Szczecin, Powstańców Wielkopolskich 72/18, 70-111 Szczecin, Poland; jaronola@gmail.com; 2 Department of Diagnostic Imaging and Interventional Radiology, Pomeranian Medical University, Unii Lubelskiej 1, 71-242 Szczecin, Poland; ewa_gabrysz@wp.pl; 3 Department of General and Interventional Radiology and Neuroradiology, Wroclaw Medical University, M. Curie-Skłodowskiej 68, 50-369 Wrocław, Poland; joanna.bladowska@umed.wroc.pl

**Keywords:** impacted third molar, panoramic radiography, cone-beam computed tomography, three dimensional printing, 3D printing, oral surgery

## Abstract

The development of radiology, rapid prototyping techniques, and the increasingly common use of 3D printing in dentistry inspires the use of these techniques to improve diagnostic and therapeutic processes. This study aimed to conduct a retrospective comparative analysis of dental panoramic radiographs, cone-beam computed tomography, and 3D printing in preoperative assessment of the procedure’s difficulty. Thirty clinical cases with a high degree of difficulty were selected, and based on evaluation with CBCT, a virtual 3D model of the region of surgical procedure was created, which was then printed using a 3D printer. The comparative analysis included the linear measurements performed in dental panoramic radiographs, cone-beam computed tomography, and 3D models in a preoperative assessment of the degree of retention and difficulty of impacted mandibular third molars in the mandible. Linear measurements performed on dental panoramic radiographs were significantly lower than in cone-beam computed tomography and 3D models. No statistically significant differences were obtained in linear measurements between 3D models and cone-beam computed tomography images except for the measurement of the lingual lamina thickness; however, due to the insignificant differences in measurements, with a mean of only 80 µm, the elective procedure of removal of the impacted third molar in the mandible may be safe.

## 1. Introduction

The qualification for removing impacted mandibular third molars is preceded by anamnesis and a physical examination of the patient including additional examinations such as radiographic diagnostics. Asymptomatic impacted third molars are often detected incidentally on panoramic radiographs taken routinely during the planning of general dental treatment. X-ray diagnostics allow for a proper diagnosis to be made and the treatment course to be determined. The most common method used to diagnose the presence of impacted third molars is panoramic radiography [1]. An accurate assessment of the morphology of impacted third molars based on a panoramic radiograph may be complicated due to the conditions of the layered radiographs. Anatomical details of impacted wisdom teeth are visible if they do not project to other nearby anatomical structures and are within the imaging layer [2]. The surgical extraction of an impacted mandibular third molar is one of the most complicated procedures in the field of oral surgery and is associated with the risk of postoperative complications [3].

In the post-war period, the invention of panoramic radiography has allowed for the significant expansion of diagnostic possibilities in dentistry [4]. Even though the highest possible precision of the panoramic radiographs (OPG) is maintained, some disadvantages of this technique are inevitable. The radiation dose in this method is approximately 14.2–24.3 µSv [5].

The discovery of cone-beam computed tomography (CBCT) has made it possible to visualize the obtained digital image in the axial, frontal, and sagittal dimensions and obtan trans-sections (i.e., perpendicular to the tangent of the dental arch) and three-dimensional reconstructions. Its fundamental feature is the ability to obtain the third dimension—the depth of the image and the non-overlapping of adjacent anatomical structures [6]. CBCT is a method that allows for obtaining spatial images at a higher resolution than images obtained with traditional CT and at a lower dose of radiation. The radiation values for the above methods are 30–1073 µSv for CBCT and 180–2100 µSv for conventional CT [7,8,9,10,11]. Three-dimensional model printing is mainly applied in oral surgery, prosthodontics, orthodontics, maxillofacial surgery, and other medical fields [12].

Many studies are available in the literature presenting the problem of retention of mandibular third molars. Radiological diagnosis based only on panoramic radiographs may be incomplete due to the fact of its two-dimensional nature [13,14]. There is only limited data in the literature comparing the reliability of panoramic radiographs and cone-beam computed tomography images as well as the use of 3D prints in preoperative planning to remove impacted teeth [15]. OPG and CBCT comparisons of third lower molars available in the literature mainly refer to position relative to the mandibular canal [16,17]. No available studies compare cone-beam computed tomography and three-dimensional models in planning surgical procedures to remove impacted wisdom teeth.

Cone-beam computed tomography provides the ability to obtain spatial reconstructions of the examined area. It allows visualization of the case in the form of a graphic file in .STL format (stereolithography), which is a three-dimensional record of the CBCT examination that allows printing it out using a 3D printer. Additive technology, which involves creating a three-dimensional model by fusing successive layers of material (resin-photopolymer or filament) to plan surgical scenarios, is becoming increasingly common. The process of fabricating three-dimensional anatomical models begins with importing a series of CBCT images in .DICOM (high-resolution digital medical data) format. Using special software, the two-dimensional image is converted into a three-dimensional visualization. Then, after selecting specific areas of interest to the operator and creating a spatial surface model, it is exported to a file in the .STL format. Three-dimensional modeling and the resulting printout allow dedicated preoperative design in oral surgery, which enables imaging of inaccessible anatomical structures so that we can reduce the risk of surgery-related complications and recovery time. The spatial medical model prepared before the surgery allows the operator to better prepare for the operation as well as to illustrate the clinical situation to the patient, the extent and difficulty of the procedure, and to discuss the course of the procedure. Three-dimensional planning allows creating a surgical protocol targeted to the patient [15].

This topic is essential due to the increasing prevalence of surgical removal of impacted third molars in the mandible. Continuing technological advances motivate modern techniques, such as 3D modeling and 3D printing, to improve the procedure planning process. In addition, the 3D model can allow visualization of the clinical case, simulation of the procedure, and detailed presentation of the procedure to the patient before it is performed. This study aimed to compare the measurements on OPG, CBCT, and 3D models and determine whether OPG is sufficient to evaluate the difficulty of removing a mandibular third molar. In addition, comparing the accuracy of 3D models can help plan the surgical removal of an impacted tooth.

## 2. Materials and Methods

The study was conducted in the Department of Oral Surgery of Medical University after obtaining approval from the Bioethics Committee (KB–0012/270/09/18).

### 2.1. Characteristics of the Study Group

Thirty selected clinical cases were evaluated by panoramic radiographs, cone-beam computed tomography, and printed using a 3D printer with digital light processing technology (Planmeca Creo, Planmeca, Helsinki, Finland). Three-dimensional imaging was performed with a Cranex3Dx tomograph (Soredex, Tuusula, Finland), with a pixel size 0.085 mm, 7–8 mA, and 89 kV. The field of view (FOV) ranged from 6 cm × 8 cm to 15 cm × 13 cm. The CBCT images were analyzed using OnDemand3DTM Dental software (OnDemand3D Technology Inc., Tustin, CA, USA). The panoramic study had a known magnification of 1:1.1, which was included in the calculations. Linear measurements of the distance of the second lower molar from the anterior edge of the mandibular ramus, the thickness of the bone cover at the impacted tooth, and its distance from the mandibular canal were evaluated on the OPG images, the image obtained by CBCT, and the 3D prints.

### 2.2. Preparing the File for 3D Printing

The first step in creating a three-dimensional model of the operating region was to import the DICOM format data obtained from the cone-beam computed tomography (CBCT) scan. After importing the CBCT data, a three-dimensional visualization was generated. The next step was to mark the bone area using a scale analogous to the Hounsfield scale. The grayscale parameters were set to highlight pixels encompassing bone structures (values above 400 U). Then, using manual editing of individual layers, bone not included in the treatment area was highlighted in each layer. It was possible to mark an impacted tooth to show it later in the finished printout. The wisdom tooth and the mandibular canal were marked layer by layer with the ERASE tool to obtain a space in the model corresponding to the location of the structures. The model was printed with transparent resin (Surgical Guide, Planmeca Helsinki, Finland). Preferences were set separately for the two initial layers and each subsequent layer. The thickness of the initial layer was set at 0.1 mm, and its exposure time was 30 s. Each subsequent layer had a thickness of 0.06 mm, and the exposure time was 3.5 s for each layer.

### 2.3. Linear Measurements on Panoramic Radiographs

Figures illustrating the measurement process are presented in the Supplementary Materials (Figures S1–S4).

Linear analysis of the panoramic radiographs was performed in the Scanora 5.2.6 computer program (Soredex, Tuusula, Finland). Parameters that were possible to compare on panoramic radiographs, CBCT images, and 3D models made with the DLP 3D printing technique were selected for comparative analysis. Each measurement was verified three times by two investigators, and their average value was analyzed. Linear measurements were determined using the measurement tool “ruler”, a component of Scanora 5.2.6 (SOREDEXN, Tuusula, Finland)and OnDemand3DTM Dental software (CyberMed, Unit K, Tustin, CA 92780, USA).

To determine the distance of the mandibular ramus from the distal surface of the second lower molar, a vertical line tangent to the proximal surface of the mandibular ramus and a line tangent to the distally largest crown convexity of the second molar were drawn. The lines were drawn so that analogous measurements could be made on the CBCT images. These were vertical lines drawn in the sagittal plane.

The width of the crown of the wisdom tooth was determined at the point of its greatest transverse convexity.

Analogous to the measurements of the distance of the second lower molar from the anterior edge of the mandibular ramus and the crown’s width, the wisdom tooth’s immersion into the bone was measured at the height of the bone cap centrally over the crown of the impacted tooth.

Analogous to the other measurements, the distance of the impacted tooth from the mandibular canal was determined as the shortest distance of the impacted tooth from the mandibular canal.

### 2.4. Linear Measurements on Cone-Beam Computed Tomography

Figures illustrating the measurement process are presented in Supplementary Materials (Figures S5–S12).

Linear analysis of images obtained from cone-beam computed tomography was performed using the OnDemand3DTM Dental software (OnDemand3D Technology Inc., USA). The parameters were measured analogously to the measurements taken on the panoramic radiographs, making it possible to compare the results obtained in OPG, CBCT, and 3D models, extending them to include the dimensioning of the width buccal and lingual bone plate at the impacted tooth site. The following images were used to make the measurements: frontal and trans-sectional images and panoramic reconstruction obtained using a tangent line drawn parallel to the course of the dental arch in the horizontal plane. In each case, the tangent line parallel to the course of the dental arch was determined to run through the center of the crown of the second molar (or, if missing, the first molar), the impacted lower molar and the mandibular ramus.

To measure the distance of the distal surface of the second lower molar, three cross-sections were used, i.e., a frontal and a trans-sectional section, and a panoramic reconstruction obtained with the aid of a tangent line parallel the course of the dental arch. The frontal section was positioned to run through the largest convexity of the second lower molar. The line marking the transsection was then set at the level of the anterior edge of the mandibular ramus. To determine the distance of the anterior edge of the mandibular ramus from the distal surface of the second lower molar using the measuring tool “ruler”, the distance between the tangent line to the mandibular ramus and the largest convexity of the second lower molar on the frontal section was marked. The space was determined along a line parallel to the course of the dental arch, running through the center of the crown of the second molar, the impacted wisdom tooth, and the mandibular ramus.

The measurement of the crown width was performed according to the position of the impacted tooth on the frontal or trans-sectional sections in the transverse dimension at the site of its greatest convexity using the “ruler” tool. The space available for the impacted tooth was also evaluated using Pell and Gregory’s classification. It should be emphasized that the Pell and Gregory classification only determines the amount of potential space for tooth eruption, i.e., its spatial location in the mandible. This classification does not determine the possibility of tooth eruption. For the study, the author adopted the following relationships: distance 1—if the amount of space is greater than the anteroposterior dimension of the tooth crown, distance 2—if the amount of space is greater than half but not more than the anteroposterior dimension of the tooth crown, and distance 3—if the amount of space is less than half the anteroposterior dimension of the tooth crown. Distances 1, 2, and 3 were evaluated analogous to the Pell and Gregory classification (1, 2, and 3).

To assess the thickness of the bony cover of the impacted mandibular third molar on CBCT, the immersion of the wisdom tooth into the bone was measured, and the width of the bone plate on the buccal and lingual side the wisdom tooth was also assessed differently from the panoramic radiographs. Frontal and trans-sectional views, as well as a panoramic reconstruction, were used to make the measurements. The transverse and frontal lines were set so that they intersected in the middle of the crown of the impacted tooth. To eliminate measurement errors, the evaluation was performed on both the trans-sectal and frontal images. In addition, the thickness of the bone cover was determined centrally above the crown of the tooth. The distance of the impacted tooth from the mandibular canal was measured. In addition, the position of the mandibular canal concerning the impacted tooth (lingual, buccal, and below the tooth roots) was assessed.

### 2.5. Linear Measurements on Three-Dimensional Models Obtained Using the DLP Method of 3D Printing

Figures illustrating the measurement process are presented in Supplementary Materials (Figures S13–S17).

Analogous linear measurements were performed on three-dimensional models obtained in DLP 3D printing technology as on the images obtained by CBCT. The model without removed supports on the bottom surface was placed on a stable substrate. Measurements were taken using an electronic caliper (Powerfix Profi) with an accuracy of 0.01 mm. Measurements were taken between the greatest convexity of the second lower molar and the anterior margin of the mandibular ramus at the exact locations where measurements were taken on the CBCT. 3D models were cut with a plaster cutting disc in bone-covered structures at the point of greatest crown convexity at predetermined locations. Measurements were made analogous to those made on CBCT. Measurements were taken of the thickness of the bony cover over the crown of the tooth, the lingual bony plate, and the buccal bony plate at the exact locations where measurements were taken on the CBCT.

The distance of the third lower molar from the mandibular canal was measured using an electronic caliper. The model was cut with a plaster cutting disc at the smallest distance of the impacted tooth from the mandibular canal. Measurements were taken analogously as on CBCT. The analysis of the space available for the eruption of the impacted tooth was also evaluated using Pell and Gregory’s classification.

### 2.6. Methodology of Statistical Analysis

Statistical analysis was performed using the statistical package R version 3.5.2 (R Core Team 2018). Quantitative variables were described using standard measures of variability and location such as arithmetic mean with standard deviation, quartiles–Q1, Q3, and median–Q2. A significance level of *p* = 0.05 was assumed in the analysis. All *p*-values below 0.05 were interpreted as indicating the presence of significant relationships. Comparison of the values of quantitative variables in two repeated measurements was performed in the absence of normality of distribution by Wilcoxon paired *t*−test. Analysis of variance ANOVA with repeated measures was used to compare the values of quantitative variables in three and more repeated measurements when the variable had a normal distribution in the measurements; otherwise, the Friedman test was used. When statistically significant differences were detected, posthoc analysis was performed to identify significantly different groups. In the absence of normality of distribution, Wilcoxon paired *t*-tests were performed. Both in case of presence or absence of normal distribution, Bonferroni correction was applied. For binary variables, the analysis of changes in the values of qualitative variables in two repeated measurements was performed with the McNemar test or for variables more than binary with the Bhapkar test. Comparing the values of quantitative variables in 2 groups was performed using the Mann–Whitney test when the distribution was not normal.

## 3. Results

All non-significant results are presented in the Supplementary Materials (Tables S1–S3).

### 3.1. Comparative Analysis of the Distance of the Second Lower Molar from the Anterior Margin of the Mandibular Ramus on the Panoramic Radiograph, Cone Beam Computed Tomography, and Three-Dimensional Model

The results in measurements were significantly different (*p* < 0.05, Friedman test, Wilcoxon tests for Bonferroni corrected pairs) on the model and CBCT, and they were significantly higher than on OPG. Detailed results are shown in Table 1.

### 3.2. Comparative Analysis of the Amount of Space Due to the Width of the Crown of the Impacted Tooth in Relation to the Distance of the Second Lower Molar from the Anterior Edge of the Mandibular Ramus on the Panoramic Radiograph, Cone-Beam Computed Tomography, and Three-Dimensional Model

The excess or deficient space for the impacted tooth was quantified. The results in each measurement were significantly different (*p* < 0.05, Friedman test, Wilcoxon tests for Bonferroni correction bound pairs). They were significantly higher on the model and CBCT than on OPG. Detailed results are shown in Table 2.

### 3.3. Comparative Analysis of the Distance of the Impacted Tooth from the Mandibular Canal on the Panoramic Radiograph, Cone-Beam Computed Tomography, and Three-Dimensional Model

The results in each measurement were significantly different (*p* < 0.05, Friedman test, Wilcoxon tests for Bonferroni correction bound pairs). They were significantly higher on the model and CBCT than on OPG. Detailed results are shown in Table 3.

### 3.4. Comparative Analysis of the Thickness of the Bone Cover at the Impacted Tooth on the Panoramic Radiograph, Cone-Beam Computed Tomography, and the Three-Dimensional Model

Complete comparative analysis of the bone cover due to the specificity of the panoramic image, cone-beam computed tomography, and three-dimensional model could only be performed on the CBCT and three-dimensional model.

#### Comparative Analysis of Buccal Bone Plate Thickness at the Greatest Crown Convexity of the Impacted Tooth on Cone-Beam Computed Tomography and Three-Dimensional Model

The results of each measurement were significantly different (*p* < 0.05, Wilcoxon paired *t*-test). They were significantly lower on CBCT than on the model. Detailed data are shown in Table 4.

### 3.5. Comparative Analysis of the Distance of the Impacted Tooth from the Anterior Margin of the Mandibular Ramus According to Pell and Gregory on the Panoramic Radiograph, Cone-Beam Computed Tomography, and Three-Dimensional Model

#### 3.5.1. Comparative Analysis of the Distance of the Impacted Tooth from the Anterior Margin of the Mandibular Ramus According to Pell and Gregory on a Panoramic Radiograph and Cone-Beam Computed Tomography

When distance measurements (1, 2, and 3) on CBCT corresponded to 1, OPG only confirmed this in 24% of cases. As many as 72% of such distance measurements on CBCT corresponded to outcome 1, measurements on OPG corresponded to outcome 2. When distance measurements on CBCT corresponded to outcome 2, OPG only confirmed this in 20% of cases. As many as 80% of the distance measurements on CBCT corresponded to outcome 2, when the measurements for OPG corresponded to outcome 3. The described differences between the OPG and CBCT results are statistically significant (*p* < 0.05, Bhapkar test). According to Pell and Gregory, there was no case for CBCT and the 3D model corresponding to distance 3. Detailed data are presented in Table 5.

#### 3.5.2. Comparative Analysis of the Distance of the Impacted Tooth from the Anterior Margin of the Mandibular Ramus According to Pell and Gregory on Cone-Beam Computed Tomography and Three-Dimensional Model

When the distance measurements (1, 2, and 3) on CBCT corresponded to result in 1, MODEL always confirmed it. When the distance measurements on the CBCT corresponded to outcome 2, MODEL confirmed this in 60% of cases, and in 40% of cases, it corresponded to outcome 1. The described differences between the results of MODEL and CBCT were not statistically significant (*p* > 0.05, McNemar test). According to Pell and Gregory, there was no case on the CBCT and 3D model corresponding to distance 3. Detailed data are presented in Table 6.

#### 3.5.3. Comparative Analysis of the Distance of the Impacted Tooth from the Anterior Margin of the Mandibular Ramus According to Pell and Gregory on the Panoramic Radiographs and Three-Dimensional Model

When the distance measurements (1, 2, and 3) on MODEL corresponded to result 1, OPG only confirmed this in 22.22% of cases. In as many as 70.37%, when distance measurements on MODEL corresponded to a result of 1, measurements on OPG corresponded to a result of 2. When distance measurements on MODEL corresponded to 2, OPG always gave a result of 3. The described differences between the results of measurements on OPG and MODEL are statistically significant (*p* < 0.05, Bhapkar test). According to Pell and Gregory, there was no case on the CBCT and 3D model corresponding to distance 3. Detailed data are presented in Table 7.

## 4. Discussion

Due to the panoramic radiography’s inability to accurately represent the curves of the dental arch and its two-dimensionality, it can distort the image obtained and overlapping of anatomical structures [18]. Despite panoramic radiography being as precise as possible, some disadvantages, such as image enlargement, image distortions (more visible in the horizontal than vertical plane), layering of the examination (only a specific layer is legibly presented in the case of lateral sections about 2 cm, in the anterior section approximately 1 cm deep into the tissues) and a high predisposition to artifacts and secondary shadows resulting from the projection of anatomical structures on the imaged area [19]. The mandibular bone anatomy in three dimensions can be depicted by cone-beam computed tomography. The proximity of anatomical structures such as the lingual nerve or the inferior alveolar nerve increases the difficulty of the procedure due to the possibility of their damage during surgical removal of the wisdom tooth, which should be considered during preoperative planning. Knowing the mandibular alveolar process dimensions in the area to be treated allows precise control of the procedure. It reduces the risk of damage to surrounding structures. In scientific publications, we can only find an analysis of data on the dimensions of the alveolar process and the alveolar region of the mandible in the context of implant treatment planning, especially on the edentulous process [20]. The limited data in the literature regarding bone coverage of the impacted tooth prompted the authors to analyze this issue. In the literature, comparisons between classical imaging of mandibular impacted teeth based on panoramic radiography and their images on cone-beam computed tomography by the authors of the publications are becoming more frequent. These comparisons are most often made longitudinally to the dental arch or evaluate only the positions of the impacted tooth relative to the mandibular canal [19,21,22,23,24,25,26,27,28].

Furthermore, only a few publications on 3D printing in the preoperative diagnosis of impacted molars [15]. This topic is essential due to the increasing prevalence of surgical removal of impacted third molars in the mandible. Continuing technological advances motivate modern techniques such as 3D modeling and 3D printing to improve the procedure planning process. This study aims to compare the measurements on OPG, CBCT, and 3D models and determine whether OPG is sufficient to evaluate the difficulty of removing a mandibular third molar. In addition, comparing the accuracy of 3D models can help plan the surgical removal of an impacted tooth.

In our study, statistically significant differences were obtained in measurements on OPG compared with dimensions on CBCT and spatial model. In contrast, no statistically significant differences were obtained when comparing CBCT images and 3D models. This information may support the accuracy of the DLP 3D printing method and the predisposition of model segmentation. Getting consistent results for cone-beam computed tomography and DLP printing demonstrates the precision of segmentation. According to Pell and Gregory, the position of the impacted tooth concerning the mandibular ramus was evaluated. Based on the results, it can be indirectly concluded that the treatments evaluated on OPG seem to be generally more complicated than those assessed on CBCT, which is related to the changes in the actual dimensions on OPG. The values obtained in the measurements on the OPG were lower, thus increasing the class (1–3) of the distance of the impacted tooth relative to the mandibular ramus. The values of each type in Pederson’s difficulty score are higher for smaller spaces and thus rated as more difficult. Similar to the comparison between CBCT and OPG, there were statistically significant differences between the model and OPG. These results support the conclusion that the CBCT-based 3D printed model fully represents the patient’s mandibular anatomy. In our study, the shortest distance of the impacted tooth from the mandibular canal was measured. Due to the projection on the panoramic radiograph of the roots of the impacted tooth in the mandibular canal, the results in almost every case dimensioned on OPG were 0. In two cases, the tooth’s roots were at a certain distance from the canal (3.2 mm; 3.7 mm). In the literature, only the position of the roots and root apices concerning the inferior alveolar nerve is usually presented. However, this situation did not consider, for example, the mandibular third molar, which is in an inverted position. In this case, the apices were not in contact with the IAN, such as the crown. For the clinician, the most crucial consideration is the proximity of the tooth to the IAN and not necessarily the root apices. It is essential to consider the proximity of the operated area and the IAN or lingual nerve during extraction to avoid perioperative complications.

The results of our study can be related to those obtained in the literature, taking into account the strengths and weaknesses of those studies. Few publications in the literature regarding linear measurements on panoramic radiographs and cone-beam computed tomography images taken along the dental arch; the distance of the impacted tooth from the mandibular canal is most often analyzed [22,23,24,25,26,27,28]. This measurement is performed in the vertical dimension. Tang et al. compared digital panoramic radiographs and cone-beam computed tomography images based on bone measurements in the alveolar region of the mandible [27]. It was shown that the results of vertical plane measurements on panoramic images were strongly correlated with CBCT (*p* < 0.05). The results of horizontal measurements were characterized by more significant variability. Abdinian et al. compared the accuracy of linear measurements in different areas of the maxilla and mandible on panoramic radiographs and cone-beam computed tomography [28]. Measurements in the vertical and horizontal planes were compared. Of note, the highest accuracy in linear measurements on CBCT was obtained in the area from canines to molars (96.2–96.9%). Zawilska et al. presented a comparison of panoramic imaging with actual anatomical conditions in the patient’s mouth [2]. The width and length of the crown and root length of the impacted teeth were measured.

Significant differences were found in the tooth crown dimensions between its actual height and width and the data obtained from OPG. Measurements made by Tang et al. showed that short distances measured on OPG were not statistically different from those made on CBCT [27]. Abdinian et al. also found no statistically significant differences in the measurements taken on the molars, but the values obtained on CBCT had a higher accuracy than on OPG (94.1–96.2%) [28]. The differences in the results between the authors may be due to the different alignment of the impacted teeth. In the lingual setting of the wisdom tooth, the projection dimension of the tooth will be larger than in the buccal site. This is due to the closer position of the impacted tooth to the detector, which makes the tooth appear smaller. This phenomenon may explain the differences in tooth size [29]. Two reports were found comparing OPG and CBCT, which evaluated the amount of space needed for the impacted tooth to erupt, but Pell and Gregory’s classification was only for panoramic radiographs [30,31]. Cook and co-authors measured the lingual bone plate of the mandibular alveolar process on cadavers and compared them with the results obtained from CBCT [32]. They demonstrated a statistical relationship between the measurements. Timock et al. investigated the accuracy of measuring buccal bone plate thickness on CBCT and its original thickness measured on cadavers [33]. They found no statistically significant differences between measurements on CBCT and direct measurements of the bone. That allows them to conclude that CBCT is a faithful representation of the patient’s human mandibular anatomy. Rodriguez et al. determined the relationship of the wisdom tooth root distance to the mandibular canal. The measurement methodology was not presented in the paper [34]. The authors only presented information on whether the roots contact the mandibular canal without providing numerical distance values. Results were presented indicating no significant relationship of root contact with the mandibular canal in OPG and CBCT.

The proximity of anatomical structures, such as the lingual nerve, increases the difficulty of the procedure due to the possibility of their damage during surgical removal of the wisdom tooth, which should be considered during preoperative planning. Knowing the mandibular alveolar process dimensions in the area to be treated allows precise control of the procedure. It reduces the risk of damage to surrounding structures. In scientific publications, we can only find an analysis of data on the dimensions of the alveolar process and the alveolar region of the mandible in the context of implant treatment planning, especially on the edentulous process [35]. Wesemann et al. conducted a study in which the accuracy of intraoral, laboratory, and CT scanners was measured using 3D printing [34]. The results indicated the highest accuracy of the 3D model made from the. STL file obtained from the cone beam CT scan. However, the authors did not consider the accuracy of 3D printing. They assumed that the printout would be a faithful copy reproducing the scanned data. The authors produced the prints using a printer working with SLA technology.

One congressional report deals with linear measurements of 3D and CBCT models of the mandible [35]. However, this study was performed on autopsy specimens. Preliminary results presented by the authors in the abstract show no statistically significant differences between model and CBCT measurements. The results obtained in the Santana et al. study highlight the potential pitfalls associated with the high inconsistency between CBCT image interpretation and the steps required to generate a 3D printed model segmentation and post-processing of the final .STL file [36]. Unfortunately, there are few reports in the literature regarding 3D printing in planning the surgical removal of an impacted tooth. The increasing access to 3D printing technology and the prevalence of radiographic 3D imaging prompted the authors to address this research topic. Comparisons and evaluations of 3D printing derived from CBCT with the patient’s anatomical conditions and assessing the accuracy of 3D printing allow this technology to be used successfully in preoperative planning [36,37,38,39]. The spatial model provides a spatial representation of a selected clinical case, facilitating the passage through the diagnostic and treatment process for both the physician and the patient. The operator can present the clinical situation to the patient before the procedure. The patient is more aware of the procedure before signing the consent. In addition, the operator can simulate the surgery before the procedure. See the spatial position of the impacted tooth concerning surrounding anatomical structures, such as the IAN. In addition, 3D printing technology makes it possible to make a surgical guide to facilitate the procedure.

To improve the diagnostic and therapeutic process and obtain better predictability of treatment results, it is reasonable to perform three-dimensional studies (CBCT) in cases of impacted mandibular third molars with a high degree of difficulty in surgical removal. 3D printing of the three-dimensional model perfectly reproduces the surgical situation and allows visualization of the clinical case. Three-dimensional printing of a model of the patient’s mandible will enable one to present the clinical problem to the patient and allow the procedure on the model in advance. It also provides the preparation of models used for medical simulation for training purposes for the operator, which could reduce complications. A three-dimensional study (CBCT) is justified for impacted mandibular third molars with a high degree of difficulty in surgical removal to improve the diagnostic and therapeutic process and obtain better predictability of treatment results. 3D printing of the three-dimensional model perfectly represents the surgical situation and allows visualization of the clinical case. This study also has some limitations. The study group consisted of only 30 patients. The 3D prints were made with only one technology. Different values could be obtained in each 3D printing technology [37]. In addition, the determination of the print area is done manually and depends on the precision of the person preparing the model. In the future, we plan to extend the study to a more extensive study group.

## 5. Conclusions

To improve the diagnostic and therapeutic process and obtain better predictability of treatment results, it is reasonable to perform three-dimensional examinations (CBCT) in the case of impacted third molars in the mandible with a high degree of difficulty in surgical removal. 3D printing of a three-dimensional model perfectly reproduces the surgical situation and allows visualization of the clinical case.

## Figures and Tables

**Table 1:** Comparative analysis of the distance of the second lower molar from the anterior edge of the mandibular ramus on the panoramic radiograph, cone-beam computed tomography, and 3D model.

Distance of the Second Lower Molar from the Anterior Edge of the Mandibular Ramu	CBCT	OPG	MODEL	*p*-Value *
Mean ± SD	14.22 ± 4.34	10.49 ± 8.36	14.58 ± 4.53	*p <* 0.001 MODEL, CBCT > OPG
Median	13.15	8.55	13.75	
Q1	11.67	6.95	11.59	
Q3	15.72	10.88	16.76	

* Non-normality of distribution in at least one measure, Friedman test + results of post hoc analysis (Wilcoxon tests for paired ties with Bonferroni correction). SD—standard deviation, Q1—first quartile, Q3—third quartile, and *p*—significance level, CBCT- Cone Beam Computed Tomography, OPG—panoramic radiograph.

**Table 2:** Comparative analysis of the amount of space due to the crown width of the impacted impacted tooth in relation to the distance of the second lower molar from the anterior edge of the mandibular ramus on the panoramic radiograph, cone-beam computed tomography, and three-dimensional model.

Amount of Space	CBCT	OPG	MODEL	*p*-Value *
Mean ± SD	1.28 ± 0.33	0.93 ± 0.68	1.32 ± 0.35	*p <* 0.001 MODEL, CBCT > OPG
Median	1.24	0.8	1.24	
Q1	1.04	0.64	1.08	
Q3	1.46	0.96	1.47	

* Non-normality of distribution in at least one measure, Friedman test + results of post hoc analysis (Wilcoxon tests for paired ties with Bonferroni correction. SD—standard deviation, Q—first quartile, Q3—third quartile, and *p*—significance level.

**Table 3:** Comparative analysis of the distance of the impacted tooth from the mandibular canal on the panoramic radiograph, cone-beam computed tomography, and three-dimensional model.

Distance	CBCT	OPG	MODEL	*p*-Value *
Mean ± SD	1.93 ± 2.29	0.23 ± 0.88	2.07 ± 2.54	*p <* 0.001 MODEL, CBCT > OPG
Median	1.05	0	1	
Q1	0	0	0	
Q3	3	0	2.96	

* Non-normality of distribution in at least one measure, Friedman test + results of post hoc analysis (Wilcoxon tests for paired ties with Bonferroni correction). SD—standard deviation, Q1—first quartile, Q3—third quartile, and *p*—significance level.

**Table 4:** Comparative analysis of buccal bone plate thickness at the greatest crown convexity of the impacted impacted tooth on cone-beam computed tomography and the three-dimensional model.

Buccal Bone Plate Thickness	CBCT	MODEL	*p*-Value *
Mean ± SD	3.24 ± 1.5	3.32 ± 1.53	<0.001
Median	3.05	3.19	
Q1	2.1	2.21	
Q3	3.95	3.98	

* Non-normality of the distribution of differences, Wilcoxon test for paired ties. SD—standard deviation, Q1—first quartile, Q3—third quartile, and *p*—significance level.

**Table 5:** Comparative analysis of the distance of the impacted tooth from the anterior margin of the mandibular ramus according to Pell and Gregory on panoramic radiograph and cone-beam computed tomography.

Distance According to Pell and Gregory								
Distance on OPG 1 and Distance on CBCT 1	Distance on OPG 2 and Distance on CBCT 1	Distance on OPG 3 and Distance on CBCT 1	Distance on OPG 1 and Distance on CBCT 2	Distance on OPG 2 and Distance on CBCT 2	Distance on OPG 3 and Distance on CBCT 2	Distance on OPG 1 and Distance on CBCT 3	Distance on OPG 2 and Distance on CBCT 3	Distance on OPG 3 and Distance on CBCT 3
6	18	1	0	1	4	0	0	0
p * < 0.001								

* Bhapkar test. *p*—significance level. 1, 2, and 3—distances according to Pell and Gregory.

**Table 6:** Comparative analysis of the distance of the impacted tooth from the anterior margin of the mandibular ramus according to Pell and Gregory on cone-beam computed tomography and 3D model.

Distance According to Pell & Gregory			
Distance on MODEL 1 and Distance on CBCT 1	Distance on MODEL 2 and Distance on CBCT 1	Distance on MODEL 1 and Distance on CBCT 2	Distance on MODEL 2 and Distance on CBCT 2
25	0	2	3
* p = 0.48			

* McNemar test. *p*—level of significance. 1 and 2—distances according to Pell and Gregory.

**Table 7:** Comparative analysis of the distance of the impacted tooth from the anterior margin of the mandibular ramus according to Pell and Gregory on the panoramic radiograph and the three-dimensional model.

Distance According to Pell & Gregory								
Distance on OPG 1 and Distance on MODEL 1	Distance on OPG 2 and Distance on MODEL 1	Distance on OPG 3 and Distance on MODEL 1	Distance on OPG 1 and Distance on MODEL 2	Distance on OPG 2 and Distance on MODEL 2	Distance on OPG 3 and Distance on MODEL 2	Distance on OPG 1 and Distance on MODEL 3	Distance on OPG 2 and Distance on MODEL 3	Distance on OPG 3 and Distance on MODEL 3
6	19	2	0	0	3	0	0	0
* p < 0.001								

* Bhapkar test. *p*—significance level. 1, 2, and 3—distance according to Pell and Gregory.

## Data Availability

The data presented in this study are available on request from the corresponding author.

## Supplementary Materials

The following are available online at https://www.mdpi.com/article/10.3390/jcm10184189/s1, Figure S1: Measurement of the distance between the parallels to the distal surface of the second lower impacted tooth and the anterior margin of the mandibular ramus on panoramic radiographs; Figure S2: Measurement of the width of the crown of a wisdom tooth at the point of its greatest protuberance in transverse dimension; Figure S3: Measurement of the height of the bone cap centrally over the crown of the impacted tooth; Figure S4: Measurement of the distance of the impacted tooth from the mandibular canal; Figure S5: The frontal section was aligned with the largest crown protuberance of the second lower molar; Figure S6: The line marking the transsection was set at the level of the anterior margin of the mandibular ramus; Figure S7: Distance between the parallel to the mandibular ramus (marked with a red arrow) and the greatest protuberance of the second lower molar (marked with a blue arrow); Figure S8: Measurement of the crown width of an impacted tooth; Figure S9: The transverse and frontal lines are aligned to intersect in the middle of the crown of the impacted tooth; Figure S10: Measurement of the lingual and buccal bone plates at the transsection; Figure S11: Measurement of the thickness of the bone cover over the crown of the tooth; Figure S12: measurement of the distance of the impacted tooth from the mandibular canal; Figure S13: Measurement of the distance of the distal surface of the second lower molar from the anterior margin of the mandibular ramus; Figure S14: Measurement of the thickness of the bone cap of an impacted third molar above its crown; Figure S15: Measurement of the width of the lingual bone plate; Figure S16: Measurement of the width of the buccal bone plate; Figure S17: Measurement of the distance between the impacted tooth and the mandibular canal on the three-dimensional model; Table S1: Comparative analysis of crown width of the impacted tooth on the panoramic radiograph, cone beam computed tomography, and three-dimensional model; Table S2: Comparative analysis of bone cap thickness above the crown of the impacted impacted tooth on the panoramic radiograph, cone beam computed tomography and three-dimensional model; Table S3: Comparative analysis of lingual bone plate thickness at the greatest crown convexity of the impacted impacted tooth on cone-beam computed tomography and three-dimensional model.
