# Supplementary material for: Correlation of Panoramic Radiography, Cone-Beam Computed Tomography, and Three-Dimensional Printing in the Assessment of the Spatial Location of Impacted Mandibular Third Molars
Source: J Clin Med. 2021 Sep 16;10(18):4189. doi: 10.3390/jcm10184189 (PMC8466116; doi:10.3390/jcm10184189)
Supplement: Supplementary file 1 [file jcm-10-04189-s001.zip › jcm-1290691-supplementary.pdf]

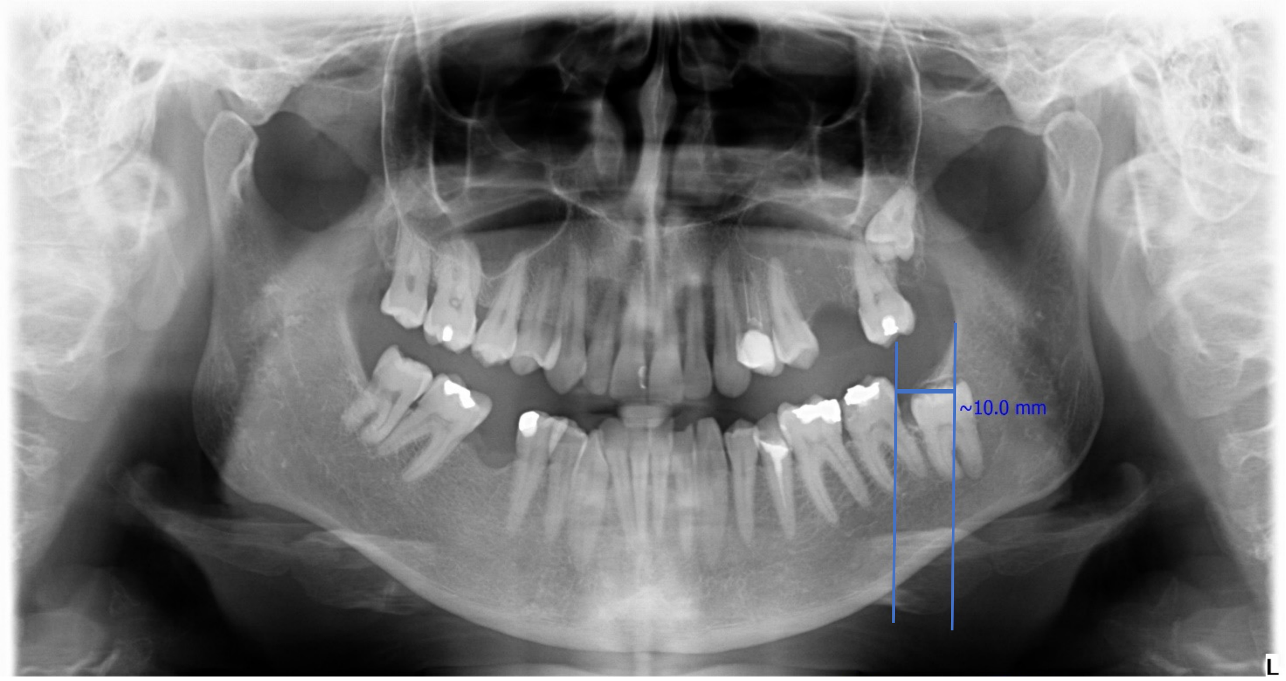

Figure S1. Measurement of the distance between the parallels to the distal surface of the second lower impacted tooth and the anterior margin of the mandibular ramus on panoramic radiographs.

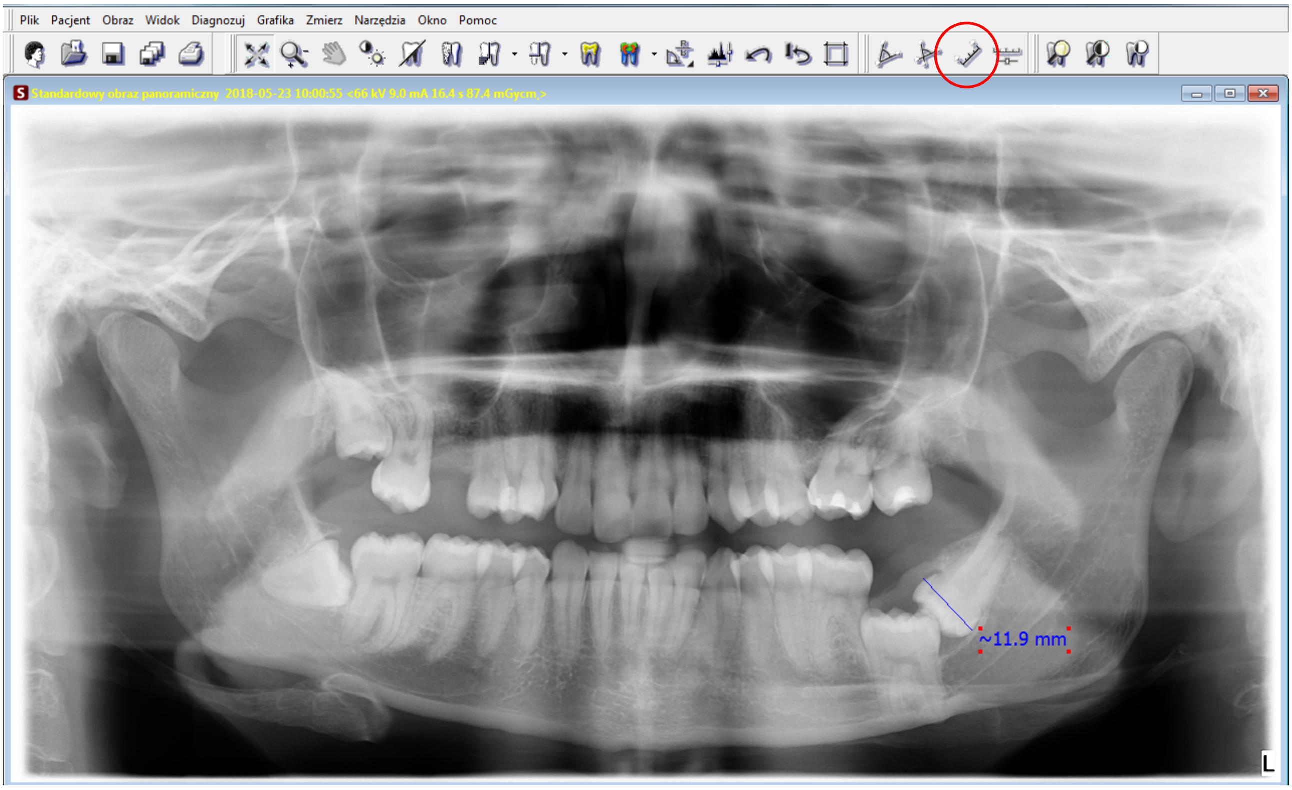

Figure S2. Measurement of the width of the crown of a wisdom tooth at the point of its greatest protuberance in transverse dimension.

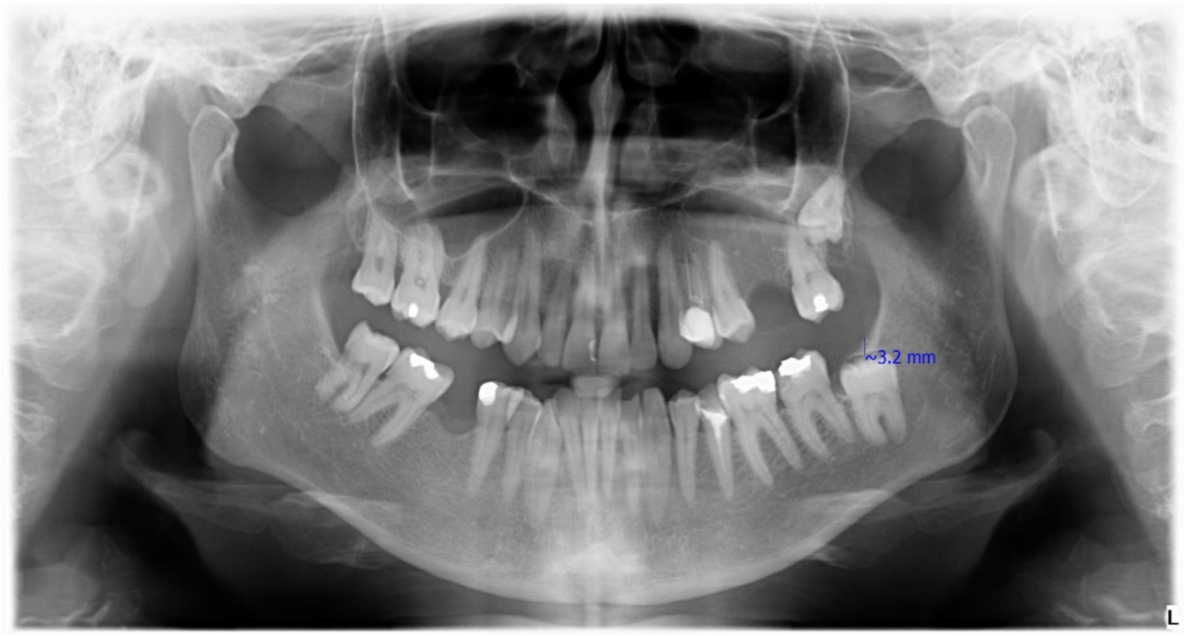

Figure S3. Measurement of the height of the bone cap centrally over the crown of the impacted tooth.

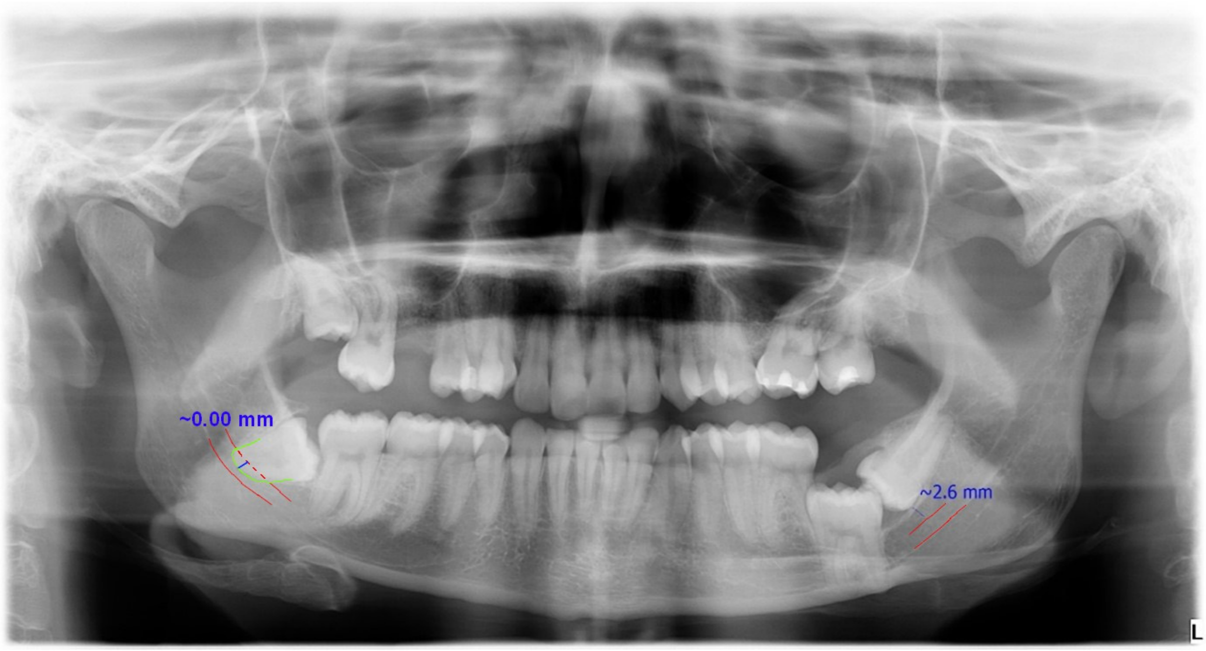

Figure S4. Measurement of the distance of the impacted tooth from the mandibular canal.

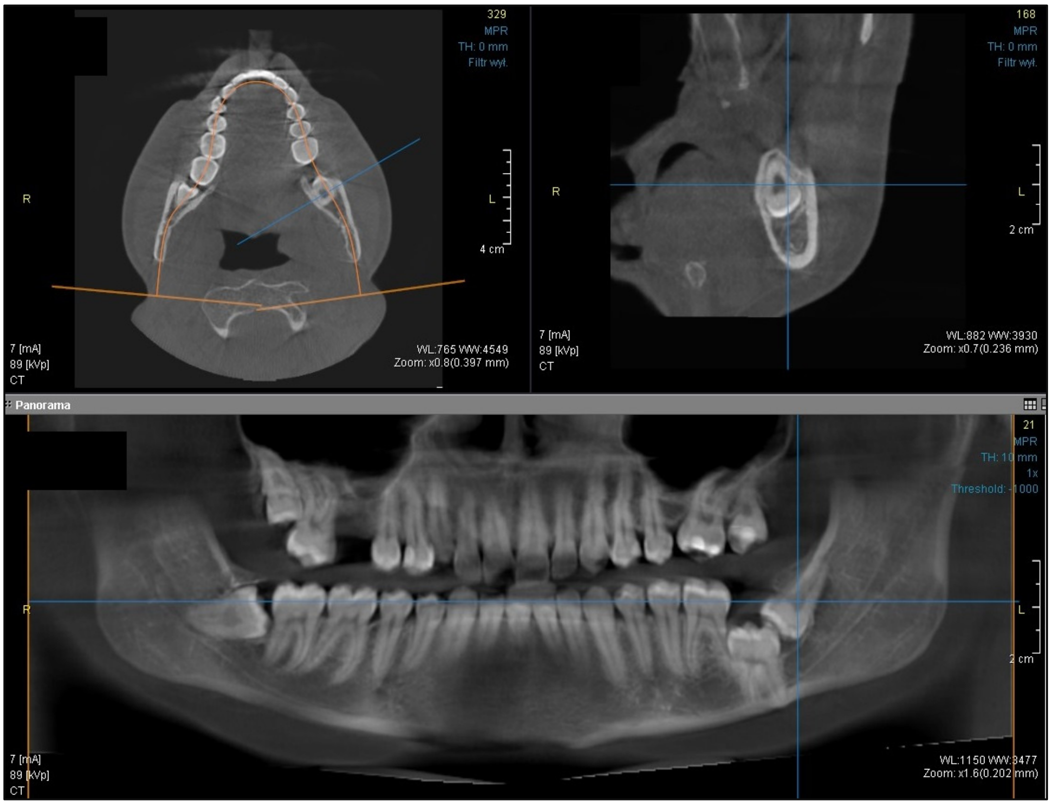

Figure S5. The frontal section was aligned with the largest crown protuberance of the second lower molar.

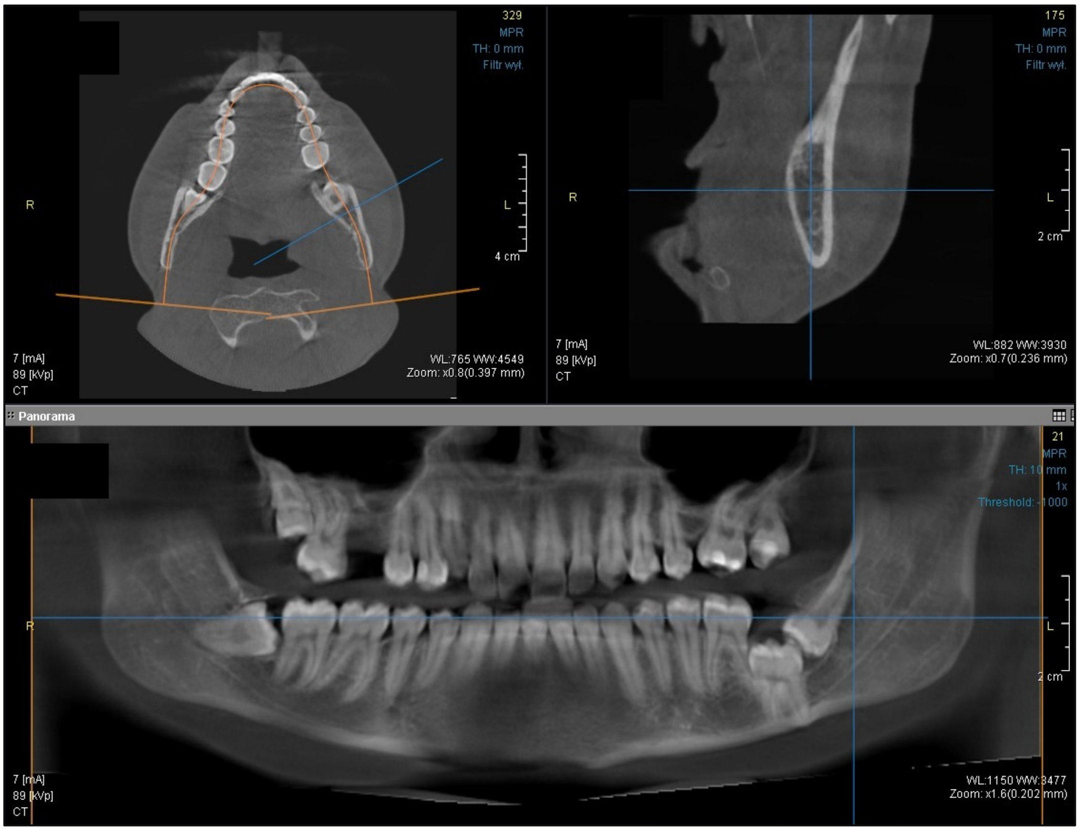

Figure S6. The line marking the transsection was set at the level of the anterior margin of the mandibular ramus.

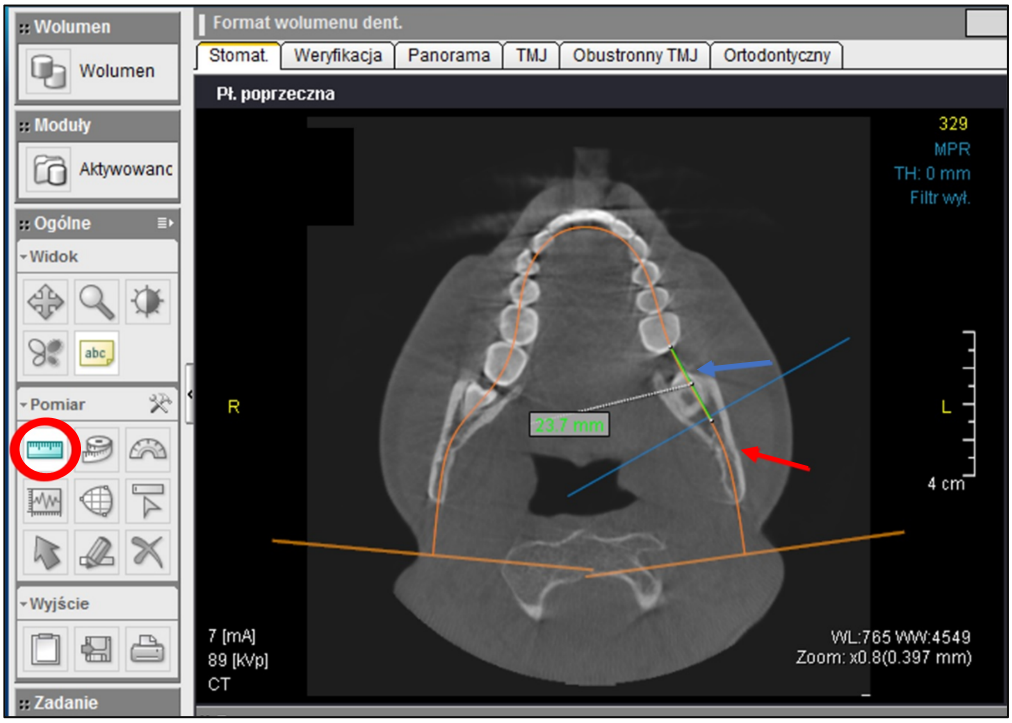

Figure S7. Distance between the parallel to the mandibular ramus (marked with a red arrow) and the greatest protuberance of the second lower molar (marked with a blue arrow).

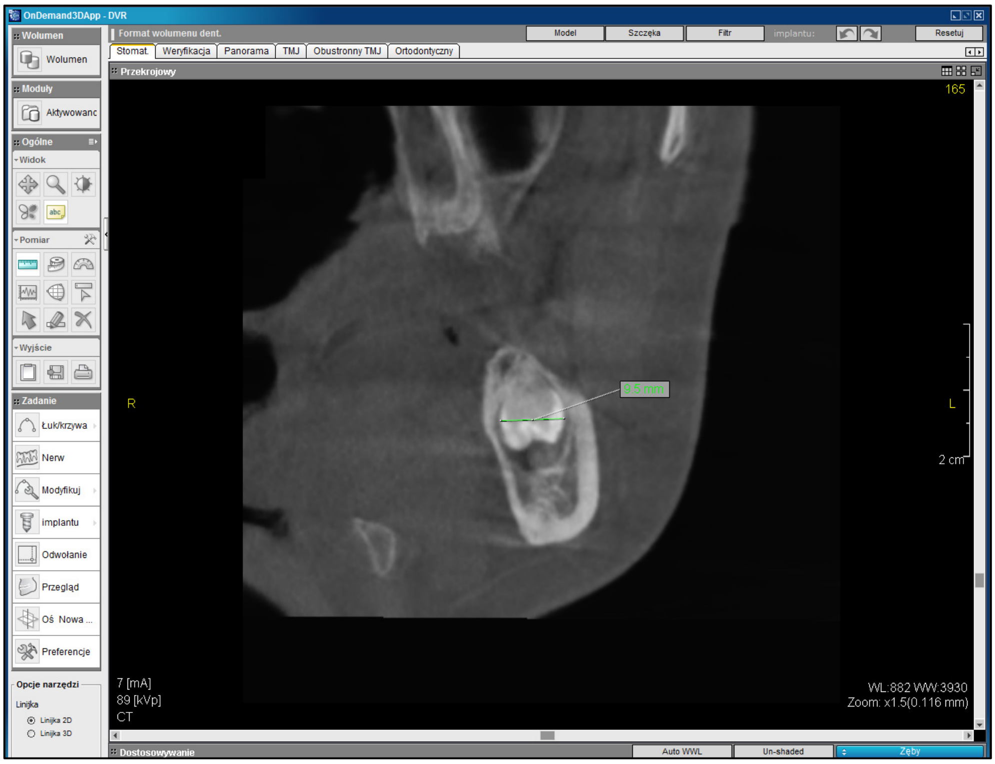

Figure S8. Measurement of the crown width of an impacted tooth.

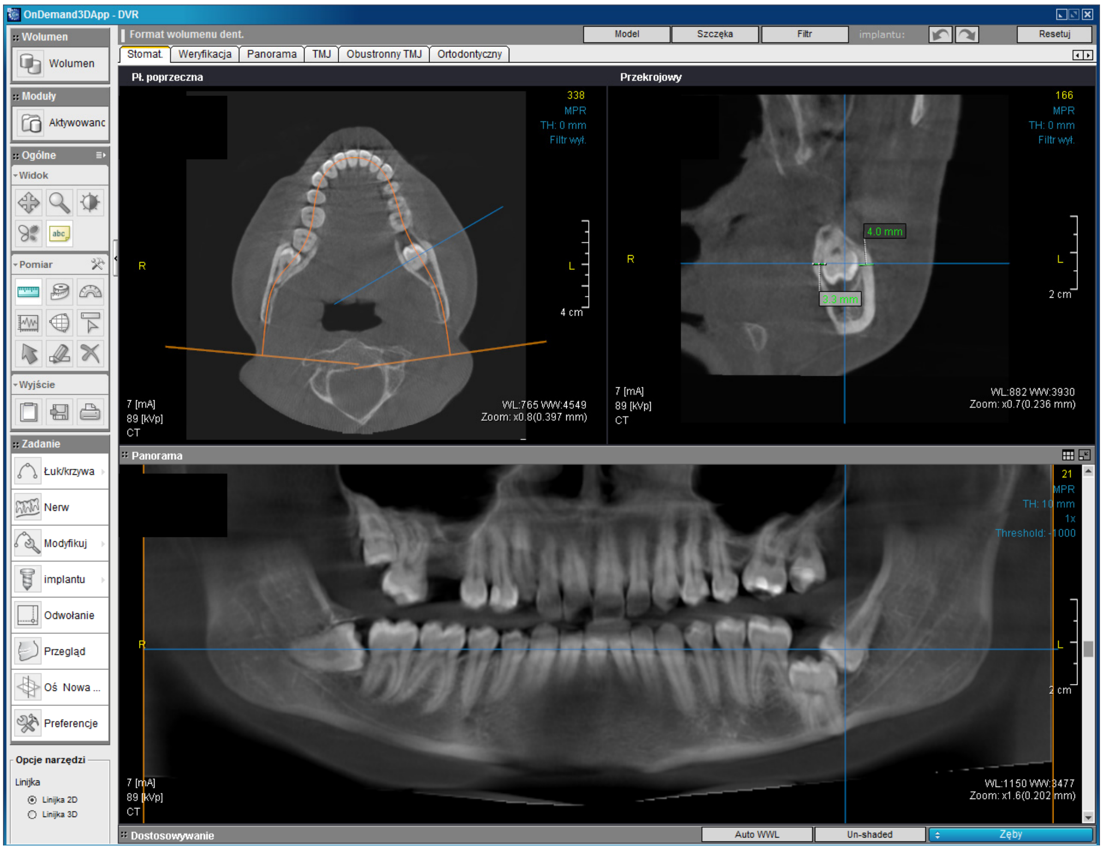

Figure S9. The transverse and frontal lines are aligned to intersect in the middle of the crown of the impacted tooth.

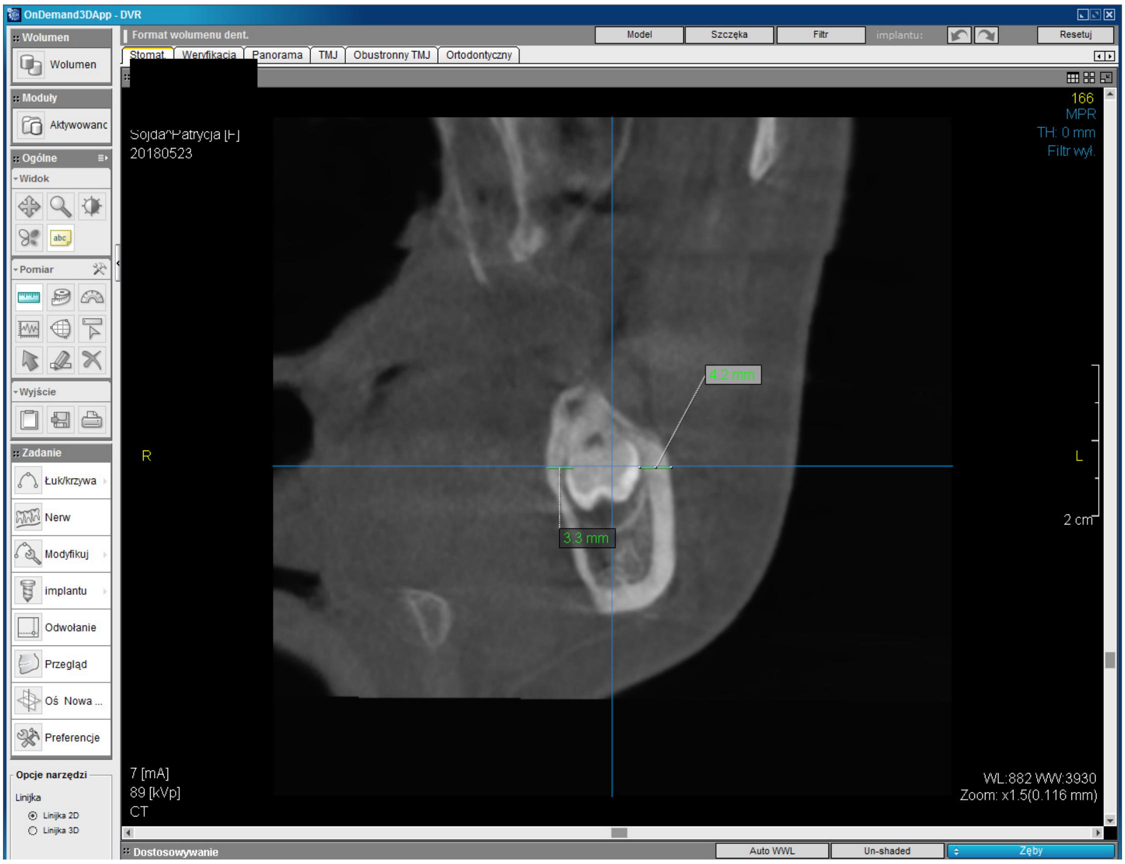

Figure S10. Measurement of the lingual and buccal bone plates at the transection.

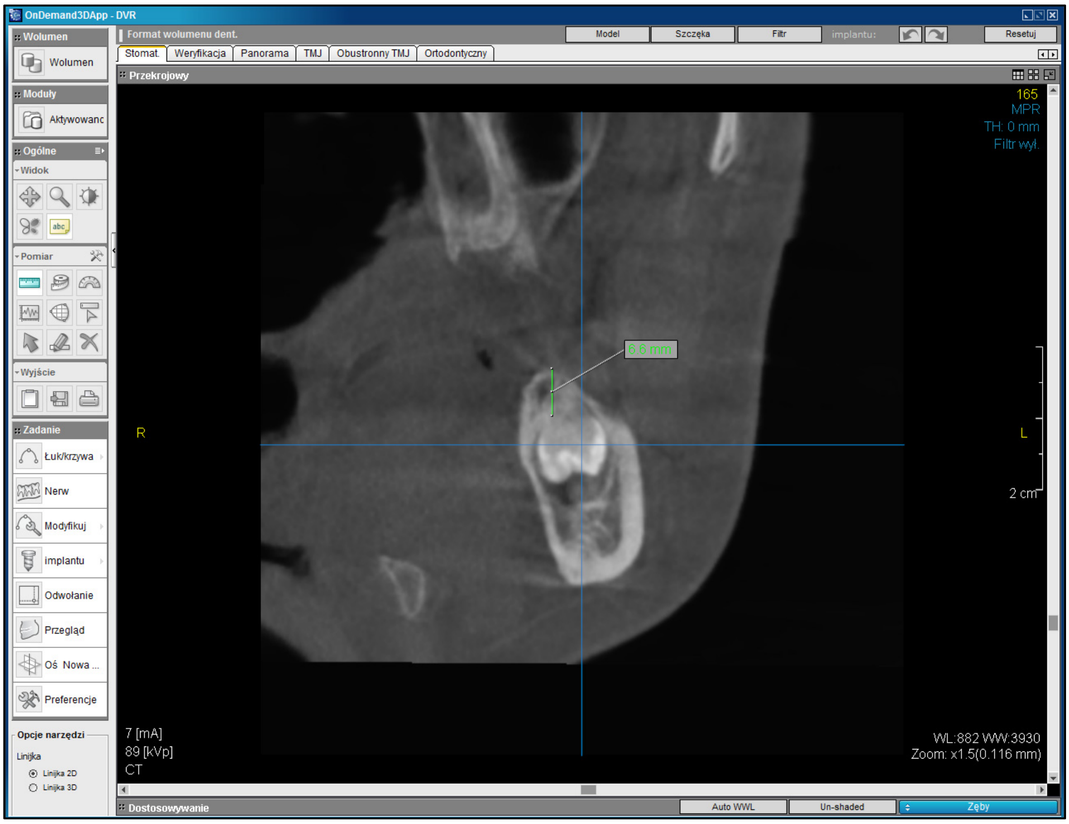

Figure S11. Measurement of the thickness of the bone cover over the crown of the tooth.

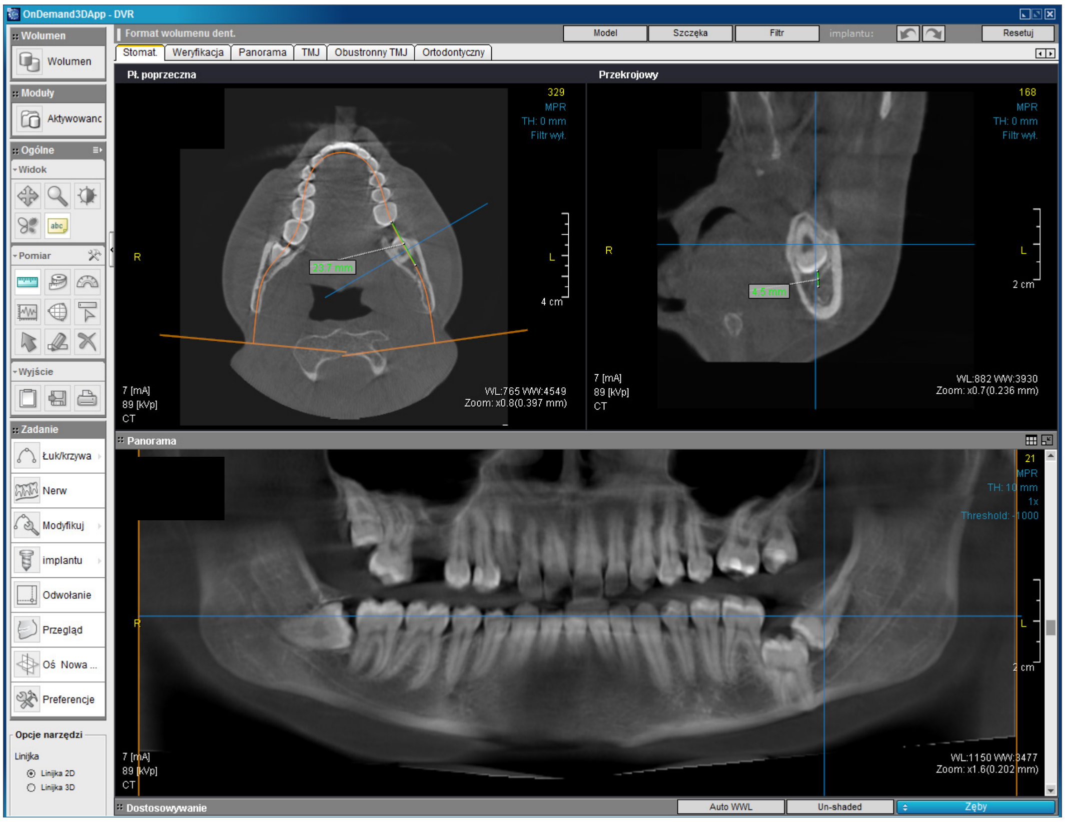

Figure S12. measurement of the distance of the impacted tooth from the mandibular canal.

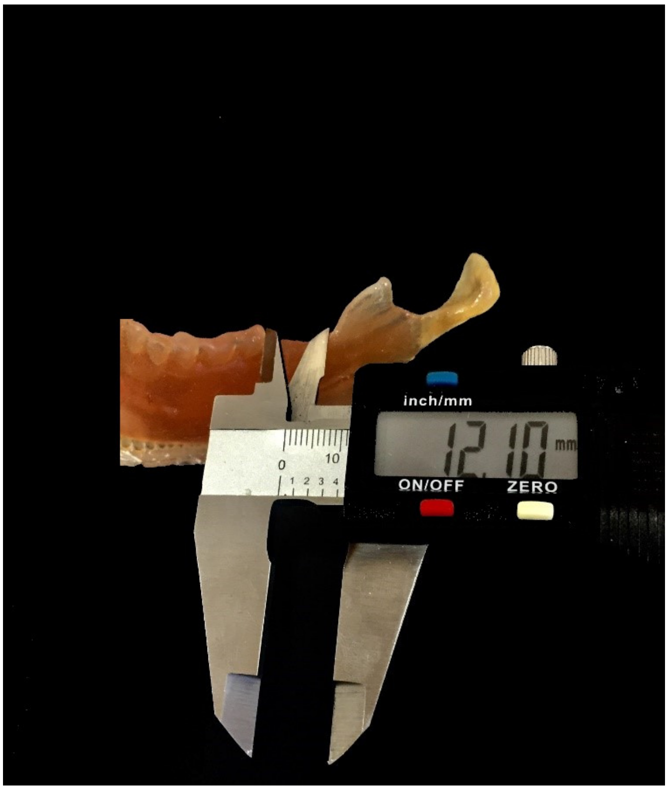

Figure S13. Measurement of the distance of the distal surface of the second lower molar from the anterior margin of the mandibular ramus.

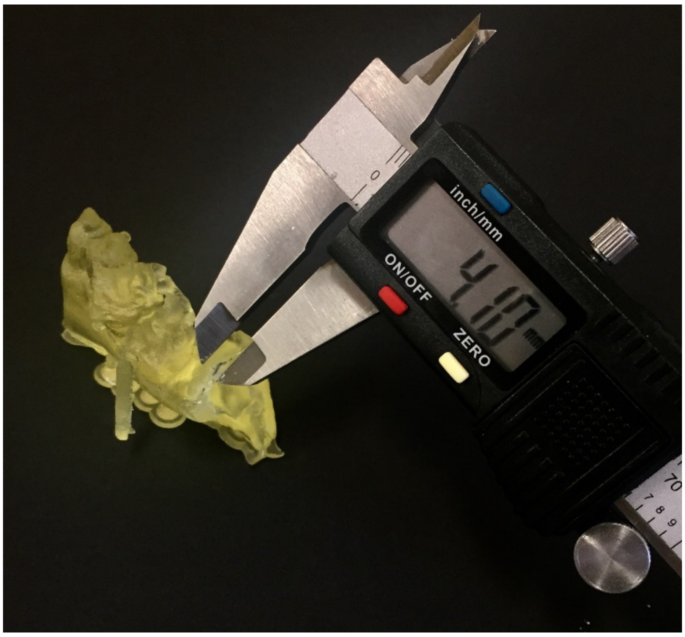

Figure S14. Measurement of the thickness of the bone cap of an impacted third molar above its crown.

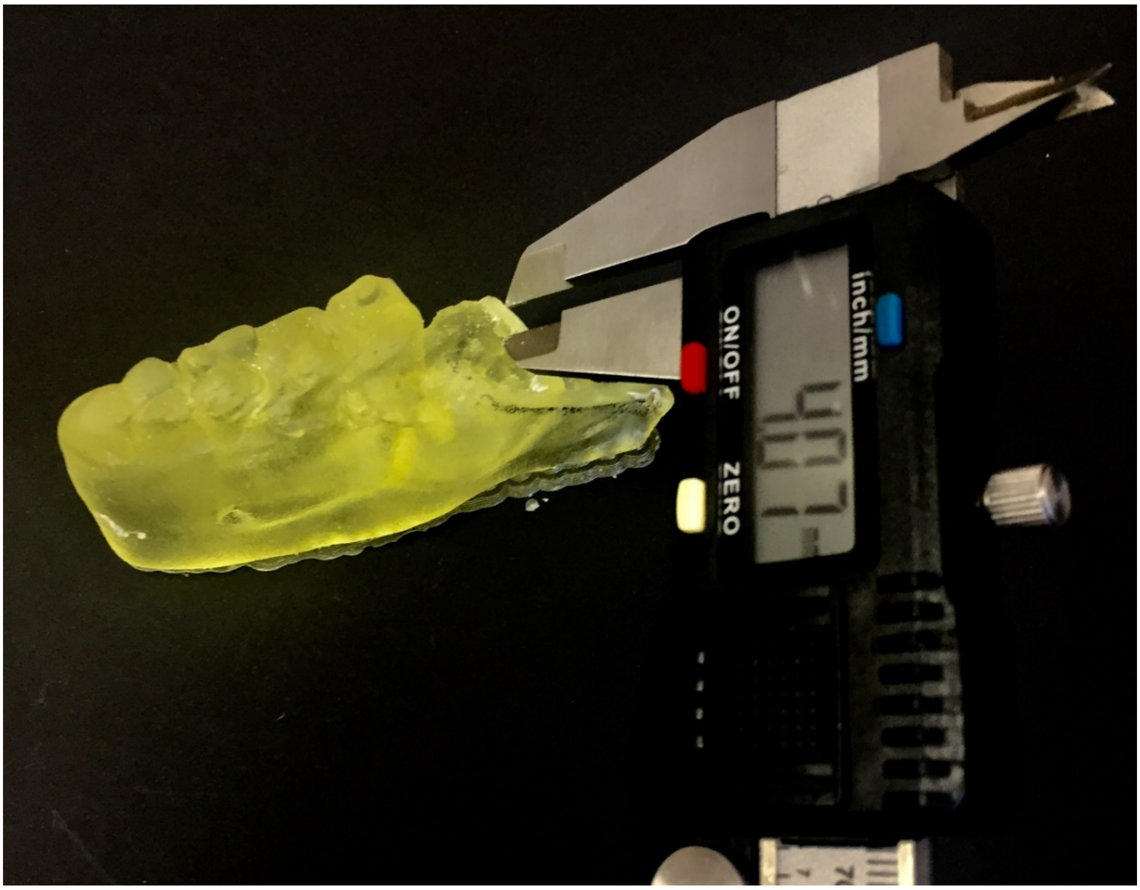

Figure S15. Measurement of the width of the lingual bone plate.

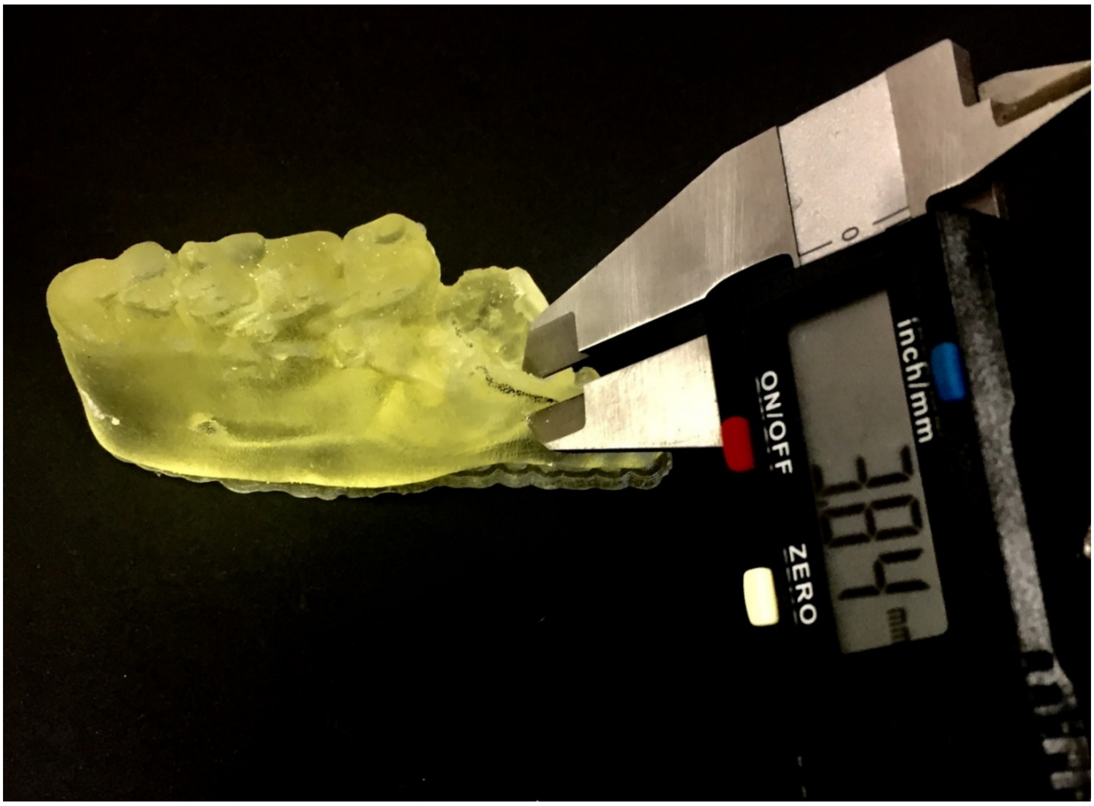

Figure S16. Measurement of the width of the buccal bone plate.

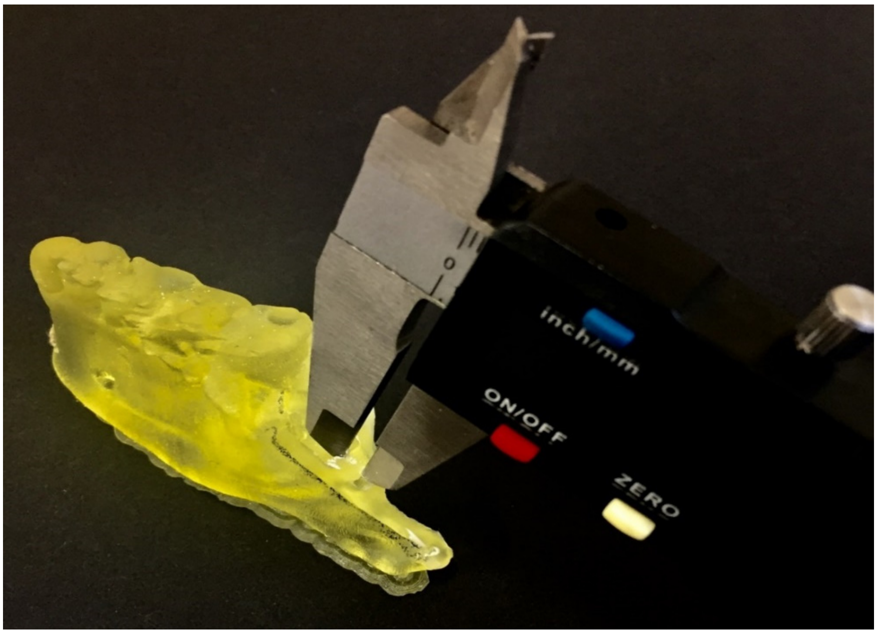

Figure S17. Measurement of the distance between the impacted tooth and the mandibular canal on the three-dimensional model.

Results:

1. Comparative Analysis of Crown Width of the Impacted Tooth on a Panoramic Radiograph, Cone Beam Computed Tomography and Three-Dimensional Model

The analysis results in each measurement were not significantly different ($p > 0.05$, ANOVA). Detailed results are shown in Table S1.

Table S1. Comparative analysis of crown width of the impacted tooth on the panoramic radiograph, cone beam computed tomography, and three-dimensional model.

Crown Width	CBCT	OPG	MODEL	*p* *
Mean ± SD	10.99 ± 1.01	11.09 ± 1.02	10.99 ± 0.97	$p = 0.542$
median	11.25	11.2	11.23	
Q1	10	10.6	10.01	
Q3	11.67	11.78	11.58	

* Normal distribution in all measurements, ANOVA with repeated measures; Explanation of abbreviations in the table: SD—standard deviation, Q1—first quartile, Q3—third quartile, *p*—significance level.

2. Comparative Analysis of the Thickness of the Bone Cover over the Crown of the Impacted Tooth on the Panoramic Radiograph, Cone Beam Computed Tomography, and Three-Dimensional Model

The results in each measurement were not significantly different ($p > 0.05$, Friedman test). Detailed data are shown in Table S2.

Table S2. Comparative analysis of bone cap thickness above the crown of the impacted impacted tooth on the panoramic radiograph, cone beam computed tomography and three-dimensional model.

Bone Cap Thickness	CBCT	OPG	MODEL	*p* *
Mean ± SD	1 ± 1.42	1.25 ± 1.61	1.04 ± 1.59	0.219
median	0	0	0	
Q1	0	0	0	
Q3	2.3	2.9	2.37	

* Non-normality of distribution in at least one measure, Friedman test. Explanation of abbreviations in the table: SD—standard deviation, Q1—first quartile, Q3—third quartile, *p*—significance level.

3. Comparative Analysis of Lingual Bone Plate Thickness at the Greatest Convexity of the Crown of the Impacted Tooth on Cone-Beam Computed Tomography and Three-Dimensional Model

The results in each measurement were not significantly different ($p > 0.05$, Wilcoxon paired *t*–test). Detailed data are shown in Table S3.

Table S3. Comparative analysis of lingual bone plate thickness at the greatest crown convexity of the impacted impacted tooth on cone-beam computed tomography and three-dimensional model.

Lingual Bone Plate Thickness	CBCT	MODEL	*p* *
Mean ± SD	1.58 ± 0.92	1.6 ± 0.93	0.262
median	1.6	1.64	
Q1	1.4	1.39	
Q3	1.98	2.02	

* Non-normality of the distribution of differences, Wilcoxon test for paired ties**.** Explanation of abbreviations in the table: SD—standard deviation, Q1—first quartile, Q3—third quartile, *p*—significance level.
